# In-vitro diagnostic in atopic dermatitis: Options and limitations 

**DOI:** 10.5414/ALX01549E

**Published:** 2017-04-07

**Authors:** K. Wichmann, A. Heratizadeh, T. Werfel

**Affiliations:** Abteilung für Immundermatologie und experimentelle Allergologie, Klinik für Dermatologie, Allergologie und Venerologie, Medizinische Hochschule Hannover, Germany

**Keywords:** atopic dermatitis, total IgE, specific IgE, specific IgG, microarray

## Abstract

Atopic dermatitis is a chronic inflammatory skin disease with a complex pathogenesis and different exogenous and endogenous trigger factors. One important factor is the sensitization to inhalant and/or food allergens. The detection of total IgE and specific IgE antibodies to inhalant and/or food allergens is one central aspect in diagnosing atopic dermatitis, especially if skin prick tests are not feasible. Many patients are polysensitized, but not all sensitizations are of clinical relevance. The challenge is to identify the sensitizations with clinical relevance and to initiate suitable therapeutic options. In this article we go into detail for the allergens house dust mite, pollen, food, and Malassezia sympodialis. Furthermore, the authors comment on the impact of the detection of specific IgG/IgG_4 _antibodies in the diagnosis of food allergy in atopic dermatitis. Moreover, new options in the in-vitro diagnostic will be explained briefly and their actual diagnostic significance in patients with atopic dermatitis will be highlighted. These options are the detection of specific IgE antibodies to recombinant allergens and the allergen chip.

German version published in Allergologie, Vol. 36, No. 2/2013, pp. 64-74 

## Introduction 

Atopic dermatitis (AD) is a chronic inflammatory skin disease and, together with allergic rhinitis, allergic bronchial asthma, and food allergy, belongs to the atopic diseases. Until their 6^th^ year of life, 10 – 15% of children show symptoms of AD, at least intermittently [34]. The prevalence has increased markedly over the past 5 decades. While in the 1950s and 60s the prevalence between birth and the age of 6 was 2 – 3%, it is 4 – 6% today. The first manifestation of the disease usually takes place within the first 2 years of life. Adults are less frequently affected (1.5 – 3%), but atopic dermatitis is not a rare disease in this age group either [34, 44]. 

It manifests in agonizingly itching eczemas, which also impair sleep significantly and thus can be extremely stressful for the entire family. In infants and toddlers, eczemas are mostly located at the extensor sides of the joints. Face and torso are also frequently affected. In older children (> approximately 2 years), adolescents, and adults, eczemas are mainly located at the flexor sides of the large joints as well as on the neck and around the eyes. Isolated hand and foot eczemas are also possible. In older adolescents and adults, pruritic nodules at the extensor sides can additionally occur. AD is considered a multifactorial disease. Besides genetic predisposition, various provocation factors also play a role in the manifestation and severity of the disease. A significant share of patients (50 – 80%, varying between studies) has IgE-mediated sensitizations against inhalant allergens and/or food allergens (sometimes associated with allergic rhinoconjunctivitis, allergic bronchial asthma, or clinically relevant food allergy). This form of AD is called extrinsic. In the intrinsic (not IgE-associated) form of the disease, the clinical picture can be identical, but no sensitizations are detectable. Furthermore, microbial factors, e.g., *Staphylococcus aureus* with partially superantigen characteristics, but also climatic and hormonal factors can be important. It is also well known that many patients experience an aggravation of the symptoms in stressful situations and when the skin is irritated. 

## Total IgE: significance and limitations in interpretation 

The determination of total IgE is still being used in the diagnosis of AD. Nevertheless, its relevance has become less in the past years due to its low specificity. On the one hand, total IgE is not increased in all AD patients. On the other hand, there are diseases that are associated with an increased total IgE without detection of specific IgE antibodies against inhalant or food allergens (see below). There are significant laboratory-dependent differences in total IgE concentrations. In general, the concentrations measured depend on the test procedure used so that a general value cannot be defined. Furthermore, the values also vary according to the patient’s age and gender [11, 22, 35] and are influenced by hormones, nicotine, and alcohol. 

These points make clear that the determination of total IgE alone is not an adequate marker for an atopic predisposition nor for the diagnosis of AD. 

## Clinical differential diagnoses when total IgE is increased 

Various skin diseases can clinically resemble AD and also manifest with increased total serum IgE. For instance, in parasitoses like toxocariasis, total IgE can be increased and severely pruritic eczematous skin lesions can be present. Other, also systemic, diseases with increased total IgE include HIV infection, graft-versus-host disease, or cutaneous T-cell lymphoma in its eczematous stage. 

In the following paragraphs, some important differential diagnoses are presented. The presentation of these clinical pictures aims at differentiation of AD from a clinical point of view and not at a complete description. 

### Seborrheic dermatitis

An important differential diagnosis at infant age is seborrheic dermatitis. Due to the frequently not yet detectable type I sensitizations, it cannot clearly be distinguished from AD by the lack of an increased total IgE titer or specific IgE antibodies. Typically, eczemas in this clinical picture manifest within the first 3 months of life. Eczemas are mainly located at the extensor sides and present with an oily yellowish crust. In contrast to AD, the diaper region is frequently involved. Seborrheic scalp eczema can be extremely pronounced in infants. In contrast to AD, pruritus is only rarely or not at all present in children with seborrheic eczema. 

### Allergic contact eczema

In allergic contact eczema, sensitization against a hapten leads to a T-cell-mediated immune reaction presenting with the clinical picture of an eczematous reaction. 

In some cases, this can resemble the clinical picture of AD. But in allergic contact eczema, the eczematous lesions are located at the sites of direct contact with the allergen. Frequent triggers are: ingredients of cosmetic products like fragrances or preservatives, nickel, and contents of rubber. A strong reaction can also result in what is known as a spreading reaction. This means that eczematous lesions can also occur far from the site of exposure. But also in this case, the contact sites are the most affected. The diseases AD and allergic contact eczema do not exclude each other. As patients with AD need to use body care products for their dry skin, contact allergies against the ingredients are more frequent. Therefore, in cases of therapy-resistant AD, the presence of an additional contact eczema should be considered. Experimental studies try to detect contact sensitization *in vitro* using the lymphocyte transformation test. However, this procedure is not relevant in daily practice. The only useful diagnostic measure to detect sensitization in a patient with contact dermatitis is still patch testing. The determination of total IgE or specific IgE antibodies does not play a role in this disease. 

### Microbial eczema

This disease, also known as nummular dermatitis, is characterized by chronically inflammatory, severely pruritic, coin-shaped, erythematous patches on which small papules and vesicles are grouped. Excoriations are frequent. The lesions mainly occur on the lower legs, torso, hands, and fingers, but the entire body can be affected. Atopic patients are more frequently affected so that differentiation from AD is not always easy. *Staphylococcus aureus* colonization is frequent. Therefore, not only are anti-inflammatory local therapy, phototherapy, and skin care necessary, but antibiotic therapy can also be useful. 

### Mycosis fungoides

This malignant disease belongs to the group of non-Hodgkin’s lymphomas and is an epidermotropic primary cutaneous T-cell lymphoma with low malignancy and a typical three-stage course (eczema stage, plaque stage, and tumor stage). The eczema stage is characterized by therapy-resistant, large, eczematous focuses with lichenification and low-grade desquamation. In contrast to AD, atrophy in the focal areas is frequent. In some patients, total IgE, but not specific IgE, is increased. Diagnosis is based on histology and on immunophenotyping of the tumor cells. This disease should particularly be considered in adults who did not suffer from AD during childhood and now develop therapy-resistant eczematous skin lesions. Therapeutic options are, depending on the stage of the disease: topic glucocorticosteroids, phototherapy, retinoids, extracorporeal photophoresis, systemic mono- and later poly-chemotherapies, immunomodulatory treatment, and, in rare cases, bone marrow transplantation. 

## Syndromes with increased total IgE 

### Hyper-IgE syndrome

In this disease, also known as Job’s syndrome, the eczema-like skin lesions are accompanied by papulopustular eruptions. The patients suffer from relapsing abscesses and therapy-resistant pustular folliculitides. This syndrome has an autosomal-dominant pattern of inheritance with a variable penetrance and is based on a primary immunodeficiency in the STAT3 gene, which clinically manifests in relapsing staphylococcus infections [39]. These can present as folliculitis and skin abscess, as mentioned above, but also as pulmonary infection and abscess, otitis, sinusitis, and arthritis. Furthermore, lymphadenopathies, growth disturbances, osteoporosis, and fractures occur. Total IgE is very high, but no sensitization against inhalant or food allergens can be detected. No causal therapy is available so that the patients only can be treated symptomatically. In some rare cases, bone marrow transplantation can be necessary. 

### Wiskott-Aldrich syndrome

Wiskott-Aldrich syndrome is a X-linked autosomal-recessive disease characterized by AD with hemorrhagic foci, thrombocytopenia (hematomas, purpura), and immune deficiency. The underlying reason is a primary functional disturbance of T-lymphocytes due to a mutation of the WASP gene, which can be detected by current techniques. This results in severe therapy-resistant infections, in particular, pneumonia, otitis media, meningitis, and sepsis. In ~ 12% of patients, malignant tumors occur. Death mainly occurs in the second decade of life due to infections and hemorrhages. Total IgE is increased, IgM titers are usually decreased, and IgA and IgG titers are normal or slightly increased. The only therapy options are symptomatic treatment as well as IVIG and bone marrow transplantation [26]. 

### Netherton syndrome

This autosomal-recessive syndrome is based on a defect of the SPINK5 gene on chromosome 5q32, which encodes the serine protease inhibitor LEKTI. The defect leads to an increased serine protease activity and thus to an increased degradation of desmoglein 1 due to which corneocytes are desquamated more and the barrier function of the skin is reduced [15]. On the skin, this defect first presents with eczematous skin lesions that can resemble severe AD. Later, the clinical picture of ichthyosis linearis circumlexa develops. The hair breaks off near the scalp due to an abnormality of the hair shaft (bamboo hair = trichorrhexis invaginata). Affected children tend to suffer from hypothermia, electrolyte imbalances, infections, and sepsis. Their growth is frequently impaired. Total IgE is increased, and specific IgE antibodies against food and inhalant allergens are present. Also for this syndrome, no causal therapy is available. 

### Omenn syndrome

This rare, autosomal-recessive syndrome is based on a general disturbance of the lymphocyte response due to a defect in the genes RAG1 and RAG2. Generalized eczemas with erythroderma and alopecia are present. Furthermore, patients suffer from severe infections and frequently also from hepatosplenomegaly and lymphadenopathy associated with eosinophilia and lymphopenia. Total IgE is significantly increased. If not treated, the disease leads to death. The only treatment option is bone marrow transplantation. 

Today, the determination of total IgE for diagnostic work-up of AD is only recommended in combination with the determination of specific IgE antibodies (or prick testing). In the diagnosis of atopic predisposition, specific IgE antibodies against inhalant or food allergens are considered marker reactions. Compared to skin tests, the in-vitro determination of specific IgE allows for a better evaluation of specific IgE levels. 

Specific IgE antibodies and T-cells cooperate in allergen-triggered AD. High-affinity FcƐ receptors can be found on MHC class II-positive Langerhans cells and dermal dendritic cells in the skin, to which antigen-bound IgE can bind and thus mediate IgE-mediated allergen presentation to skin-infiltrating T-cells [23]. In AD, the impact of allergic reactions always has to be evaluated for each individual case. For this purpose, blood tests (detection of specific IgE antibodies) and skin prick testing are available. The clinical relevance of sensitizations has to be investigated for each individual case using avoidance and/or challenge tests. Sensitization alone frequently does not justify avoidance or therapeutic measures. 

## For which allergens do therapeutic measures play a role if their clinical relevance has been shown? 

### House dust mite

One of the most frequently clinically relevant allergens in AD is that of the house dust mite. In addition to rhinitic and asthmatic symptoms, sensitization against house dust mite can result in aggravation of AD. Various studies on this topic have tried to investigate the effect of a reduction of house dust mite on AD. The results were inconsistent. It was difficult to compare these studies because they used different methods of avoidance as well as different carriers of allergens, e.g., pets. In one of the first studies, carried out in 1996, the activity of AD could be significantly reduced in pediatric and adult patients by avoidance of house dust mite (using encasing mattresses and pillows as well as acaricide sprays) [38]. Encasings in combination with the use of special vacuum cleaners also led to mite reduction and to improvement of symptoms in children [32]. Holm et al. [18] could show that patients who were not sensitized or exposed to house dust mites also benefitted from encasing measures. This puts the focus on other allergens, superantigens, or irritants. In the same year, a study on adults was published in which encasing measures led to a reduction of allergens but not to symptom improvement [19]. Similar results were obtained in another study published 1 year later [28]. Due to the partially positive results obtained in clinical trials and to the pathophysiologic concept, the current guideline recommends encasing measures for patients with AD and sensitization against house dust mite [41]. 

Another possible therapeutic option is specific immunotherapy (SIT). It is the treatment of choice in airway symptoms (allergic rhinoconjunctivitis and allergic bronchial asthma) caused by the house dust mite allergen. Until a few years ago, AD was a contraindication for SIT. It was suspected that, although the airway symptoms might improve, the AD could worsen. A review paper published in 2006 could identify five studies that had a placebo-controlled design and therefore could be compared with each other [8]. In all of these studies, a beneficial effect of SIT on AD could be demonstrated. In all other studies investigated (case reports, observational studies), no dramatic aggravation of AD could be observed under SIT. Original data that have so far only been presented at meetings (EAACI 2001 in Istanbul) show that the subgroup of severely affected AD patients could probably benefit the most from SIT [27]. Further multicenter, placebo-controlled trials with well-characterized patients are necessary to investigate the role of SIT in AD therapy. 

### Food allergy and atopic dermatitis

Patients with AD can concomitantly suffer from food allergies. Up to one third of children with severe AD and a minority of adult AD patients are affected [16, 42]. In infants and toddlers, staple foods play the most important role as triggers of food allergy; in adults, birch pollen-associated foods are of particular importance. The latter can also be relevant in pediatric patients [7]. Food allergy in AD patients can clinically present in the form of immediate-type or delayed-type reaction [1]. In immediate-type reactions, erythemas, urticaria, and angioedemas can occur, but not eczemas. Furthermore, bronchial, gastrointestinal, and cardiac symptoms of anaphylaxis can occur within minutes after ingestion of the food in question. However, some patients also show an isolated delayed-type reaction; this frequently only manifests itself 6 hours to 2 days after ingestion of the food through flare-ups of the pre-existing eczemas. There are also hybrid forms in which symptoms of immediate-type as well as delayed-type reactions occur. More than half of the AD patients with concomitant food allergy show eczemas, either occurring alone or together with immediate-type reactions [6]. It has been shown that patient history has a very good predictive value for diagnostic work-up of food allergy if an immediate-type reaction is present, but only a 30% positive predictive value when a delayed-type reaction occurs. The diagnostic work-up of food allergy – particularly of allergic delayed-type reactions – is still complex. The gold standard for diagnostic work-up of food allergy (in AD patients) is still double-blind, placebo-controlled, oral food challenge (DBPCFC) [43]. Carrying out a DBPCFC entails high personnel expenditure at the hospital and is also very time-consuming for the patient. This could be the reason why there are more scientific studies on IgE-mediated sensitization to food while the clinical relevance of these sensitizations remains less investigated. In this context, the identification of possible predictors for the development of food allergy in AD is also of major importance. 

Under these circumstances, how important is the measurement of specific IgE? Formerly, cut-off values were used for pediatric patients in order to avoid complex and sometimes even dangerous oral challenge tests [33]. However, in this study, only 61% of children suffered from AD, and oral challenge testing had not been carried out in all children [33]. Furthermore, the values measured could not be confirmed in subsequent studies [9, 25]. 

In an international cohort study investigating 2,184 children, it was be demonstrated that high specific IgE titers (i.e., “high-risk” IgE titers, defined according to the specific food) against cow’s milk, hen’s egg, and/or peanut were most frequently observed in infants in whom eczema had manifested before the end of their 3^rd^ month of life [17]. When eczema manifested after the 1^st^ year of life, the prevalence of these high-risk IgE titers was lowest. Furthermore, children with high-risk IgE titers suffered from particularly severe forms of AD. The earlier an eczema manifests in children and the more severe this eczema is, the more frequently extremely high IgE sensitizations against food can be observed. To be able to make certain recommendations for the diagnosis and handling of such high-risk IgE titers, further investigation of the clinical relevance in this high-risk group would be desirable. 

Kjaer et al. [20] provided interesting insights into the association between an earlier IgE sensitization and the development of an allergic disease at the age of 6 years. The earlier occurrence of IgE sensitization against foods was significantly associated with the presence of AD and bronchial asthma at the age of 6 years. In addition, most of the children in whom sensitization was present at the age of 6 years and who suffered from AD, bronchial asthma, or allergic rhinoconjunctivitis, had already been sensitized against foods at the age of 6 months. According to this, an early sensitization against foods could be a marker for the risk of the manifestation of a later allergic disease. In this birth cohort study, a 6-year incidence of food allergy of 3.7% (20/534) based on food challenge testing could be demonstrated [13]. In 18 of these 20 children, the diagnosis AD was made at least once within this period of time. This means that almost all children in whom food allergy was diagnosed also suffered from AD. 

Despite these current findings, oral food challenge testing is still necessary in daily practice in order to confirm the diagnosis of food allergy in AD patients. The importance of this diagnostic procedure is also highlighted by results from a retrospective study by Fleischer et al. [14]. This study evaluated oral food challenge tests, including the assessment of possible delayed-type reactions in 125 children, adolescents, and young adults (median age: 4 years), of whom 96% suffered from AD. The study aimed at assessing the number of cases in which a previously administered elimination diet was actually justified. The evaluation of food challenge testing showed that food allergy in AD had been over-diagnosed, as 84 – 93% of foods could be re-introduced into the diet after the food challenge test. Earlier studies have clearly demonstrated that 50 – 80% of children who suffered from allergy against cow’s milk or hen’s egg in early childhood, developed tolerance to this food by school age. The finding that food allergy seems to be less frequently symptomatic the older a patient is might contribute to the fact that only few studies on food allergy in adults are available. In Northern and Central Europe, most adults suffering from food allergy have an underlying sensitization against birch pollen. This birch pollen-associated food allergy could be identified as a trigger factor of pre-existing AD in a subgroup of adults [30]. Results from a German questionnaire-based study in 1,739 non-selected participants from the general population confirm that food allergies are markedly less frequent in adults than in children, independently of AD [44]. In 28 patients (1.6%), the diagnosis AD could be confirmed. Of these 28 patients, only 4 suffered from food allergy as diagnosed by oral food challenge. In another study using a similar design, an increased risk of food allergy could be identified for women (point prevalence 3.3%) as compared to men (point prevalence 1.8%) [36]. 

## Significance of specific IgG and IgG_4_ antibodies in AD 

Several manufacturers offer solutions for detecting food-specific IgG or IgG_4_ antibodies, which claim to be useful in the diagnosis of food allergy, food intolerance, or other possibly food-related diseases. Although a few studies on this topic exist, some of even placebo-controlled [2, 3], they cannot be considered a proof of validity due to their disputable methods and evaluation approaches. In healthy individuals, IgG_4_ reflects contact with an allergen without being associated with allergic reactions [40]. Thus, it is not possible to distinguish between “healthy” and “ill” by measuring specific IgG_4_ antibodies. The formation of IgG_4_ is part of an immunologic, not a pathologic, reaction. This illustrates that IgG_4_ is also inadequate to diagnose non-immunologically mediated food intolerance. Scientific associations covering the specialties allergology (DGAKI, EAACI), internal medicine (DGIM), laboratory medicine (DG-KL), nutritional medicine (DAEM), and ecotrophology (VDOE) have taken a firm stand and state that, “the determination of IgG and IgG_4_ antibodies against food has no diagnostic value” [4, 21, 24, 37]. 

## Recombinant allergens and microarray (allergen chip) 

Recombinant allergens are produced using biotechnological procedures and correspond to single components of allergens from an allergen source, e.g., Bet v 1 is an allergen from birch pollen extract. Unlike allergen extracts, recombinant allergens have a consistent quality due to a standardized manufacturing process and thus can be well quantified and standardized [31]. Allergen extracts, on the other hand, can vary between manufacturers, but also between batches from the same manufacturer. In the diagnostic work-up of AD, the determination of specific IgE antibodies is only significant in very special situations. For example, in patients with pollen allergy, by knowing the recombinant major allergen against which the patient is sensitized, further investigation on cross-reacting foods can be carried out if the patient has experienced problems with certain foods that could not be further evaluated based on patient history. 

Another new development is the microarray (allergen chip), which is now commercially available and allows for the detection of specific sensitization against currently more than 100 inhalant and food allergens. This test assesses traditional allergen extracts as well as recombinant allergens. One advantage of this test is that only very low amounts (20 µl) of blood are necessary, which is particularly desirable in pediatric allergology. In addition, it is easier to identify patterns of sensitization based on knowledge of protein families; this also provides further insight into the development of the sensitization (i.e., primary sensitization vs. cross-reaction) [5]. Certain food allergens (e.g., Ara h 2 in peanut) are also associated with severe reactions. This information is important for the patients, but it is also helpful in the planning of oral challenge tests. 

Further studies are necessary to show whether these test procedures are really useful in routine diagnostic work-up. For example, a two-step diagnosis would be conceivable: first, specific IgE is determined based on patient history, and, in a second step, a microarray is carried out to plan oral challenge testing in specialized centers [12]. 

Ott et al. [29] showed a good correlation between the CAP FEIA system for recombinant allergens and a microarray for inhalant allergens in a small collective of 40 adult AD patients. Furthermore, a correlation between specific IgE against PR-10 proteins and the severity of the disease (evaluated using SCORAD) was shown in a small prospectively studied collective of 20 patients. Possibly, the microarray can also be used in future epidemiological studies to assess markers for special forms of the disease (persistent, severe manifestation of other atopic diseases). Furthermore, it is a sensible diagnostic tool to differentiate between the intrinsic and extrinsic form of AD. 

One problem is the large amount of data produced by this kind of test. In many AD patients, several sensitizations are present at the same time – many of them without having clinical significance. If this kind of test is carried out, the responsible allergist or physician needs to investigate the clinical significance of the detected sensitizations. Lack of time or experience could lead to the results being forwarded to the patient without previous identification of the significant allergens. Thus, this kind of test should only be carried out by an experienced allergist and only for very certain purposes. Before this test can be recommended for daily practice, large prospective unselected cohort studies and investigations in certain high-risk groups are necessary. 

## Malassezia sympodialis 

Sensitization against the yeast Malassezia sympodialis probably plays a role in the subgroup of AD patients suffering from the head-neck-shoulder variant of the disease. Studies have shown that Malassezia sympodialis is present in more than 90% of AD patients. Up to 67% are sensitized against this yeast, and, remarkably, some patients do not show any further kind of sensitization. Experiments showed that dendritic cells are able to absorb Malassezia sympodialis and present T cells. In most clinical studies, 2-month systemic antimycotic therapy, using ketoconazole or itraconazole, resulted in reduced IgE titers and a slight improvement of eczema, particularly in the head-and-neck variant. 

## Conclusion 

In conclusion, the determination of specific IgE antibodies can provide valuable information but is not necessarily evidentiary when food allergy is suspected. The gold standard is still double-blind, placebo-controlled, oral challenge testing. In the case of inhalant allergens, the determination of specific IgE antibodies can be indicated under certain circumstances, such as the impossibility of carrying out prick tests. One question is the indication of encasing mattresses and pillows (specific IgE antibodies against mite allergens) or when cross-allergy to birch pollen is possible. Microarrays offer interesting new aspects for the diagnostic work-up of type I sensitizations, but cannot be recommended for use in daily practice yet. 
